# Retention of visa-trainee post-graduate residents in Canada: a retrospective cohort study

**DOI:** 10.1186/s12960-021-00638-4

**Published:** 2021-08-17

**Authors:** Maria Mathews, Dania Koudieh, Yanqing Yi, Lindsay Hedden, Emily Gard Marshall, Asoka Samarasena, Geoff Barnum, Ivy Bourgeault

**Affiliations:** 1 Department of Family Medicine, Schulich School of Medicine and Dentistry, Western Centre for Public Health and Family Medicine, University of Western Ontario, 1465 Richmond Street, Second Floor, Rm 2140, London, ON N6G 2M1 Canada; 2 Canadian Post-M.D. Education Registry, Association of Faculties of Medicine of Canada, 2733 Lancaster Road, Suite 100, Ottawa, ON K1B 0A9 Canada; 3 Division of Community Health and Humanities, Faculty of Medicine, Memorial University, 300 Prince Philip Drive, St. John’s, A1B 3V6NL Canada; 4 Faculty of Health Sciences, Simon Fraser University, 8888 University Drive, Burnaby, BC V5A 1S6 Canada; 5 Department of Family Medicine, Dalhousie University, 1465 Brenton Street, Suite 402, Halifax, NS B3H 4R2 Canada; 6 Department of Anesthesia, Faculty of Medicine, Memorial University, 300 Prince Philip Drive, St. John’s, NL A1B 3V6 Canada; 7 School of Sociological and Anthropological Studies, Faculty of Social Sciences, University of Ottawa, 120 University Private, Ottawa, ON K1N 6N5 Canada

**Keywords:** Visa trainees, Funding, International medical graduates, Cohort study, Retrospective, Post-graduate medical education, Residency programs

## Abstract

**Background:**

Visa trainees (international medical graduates [IMG] who train in Canada under a student or employment visa) are expected to return home after completing their training. We examine the retention patterns of visa trainee residents funded by Canadian (regular ministry and other), foreign, or mixed sources.

**Methods:**

We linked data from the Canadian Post-MD Medical Education Registry with Scott’s Medical Database for a retrospective cohort study. Eligible trainees were IMG visa trainees as of their first year of training, started their residency program no earlier than 2000, and exited training between 2006 and 2016. We used Cox regression to compare the retention of visa trainees by funding source.

**Results:**

Of 1,913 visa trainees, 431(22.5%), 1353 (70.7%) and 129 (6.8%) had Canadian, foreign, or mixed funding, respectively. The proportion of trainees remaining in Canada decreased over time, with 35.5% (679/1913); 17.7% (186/1052); 10.8% (11/102) in Canada one, five, and ten years, respectively after their exit from PGME training. Trainees who remained on visas (HR: 1.91; [95% CI 1.59, 2.30]), were funded exclusively by foreign sources (HR: 1.46; [95% CI 1.25, 1.69]), and who had graduated from ‘Western’ countries (HR: 1.39; [95% CI 1.06, 1.84]) were more likely to leave Canada compared to trainees who became citizens/permanent residents, were funded by Canadian sources, or were visa graduates of Canadian medical schools, respectively.

**Conclusions:**

Most visa trainees leave Canada following their training. Trainees with Canadian connections (funding and/or change in legal status) were more likely to remain in Canada.

## Background

In August 2018, the diplomatic dispute between the Kingdom of Saudi Arabia and Canada placed a spotlight on visa trainees in post-graduate medical education (PGME) programs in Canada [1–3]. Unlike other international medical graduates (IMG) who are Canadian citizens or permanent residents, visa trainees come to Canada to train on a student or employment visa and are expected to leave Canada after completing their training. By the numbers, there is at least one first-year residency position for every medical school graduate in Canada (i.e. Canadian medical graduate [CMG]), although graduates are not guaranteed their first choice of specialty or location of training. There are also roughly 450 first-year positions available each year for permanent resident/citizen IMG [4]. These positions are funded by provincial ministries of health, and CMG and Canadian citizen/permanent resident IMG apply for admission to residency programs through the Canadian Residency Matching Service. In contrast to this system, which is highly coordinated across provinces and training sites, the number of positions for visa trainees are determined locally by the training site, with minimal, if any, coordination between sites or provinces [5, 6]. Moreover, funding for visa trainees is provided by sponsors (e.g., foreign governments, foreign and Canadian agencies) [6, 7] that must cover the trainee’s stipend, tuition and provide a fee (e.g., $100,000/year) to the training site/program [8, 9]. While the goals of the visa training program are to meet Canadian training program needs, provide services, and/or fulfill Canada’s obligation to support medical training in less developed countries [6, 7, 10], they also represent a source of revenue to PGME programs.

**Fig. 1:**
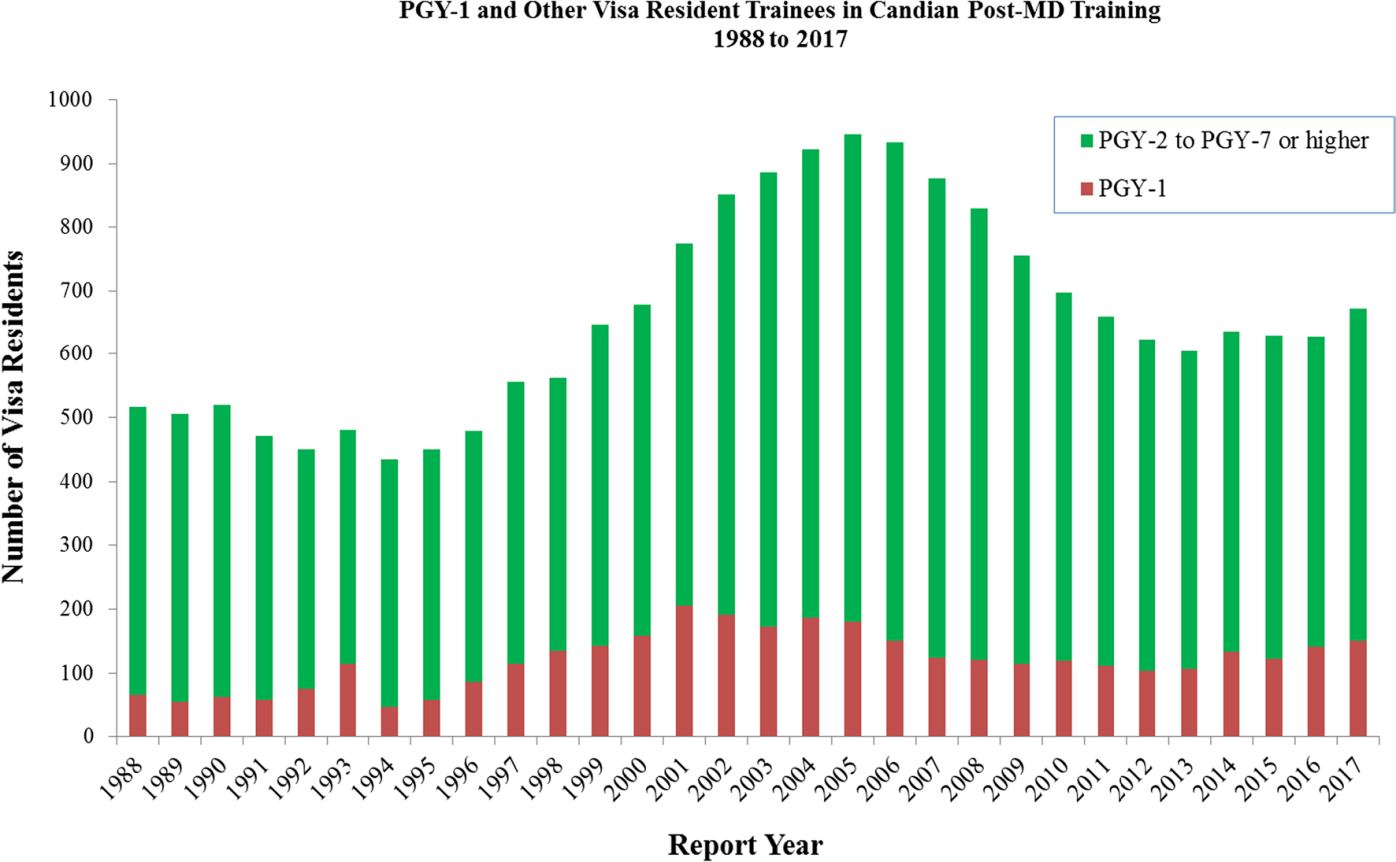
Number of visa trainees in Canadian post-graduate residency programs by year of training, 1988–2017

The number of visa trainees in residency programs has grown in the past 30 years, from 66 first year residents in 1988 to 151 in 2017 (Fig. 1). While visa trainees are expected to return home after their training, existing studies have estimated that between 19 and 52% of these residents remained in Canada roughly five years after their PGME training [7, 8]. Given the growth in the number of visa trainees if residency programs, and retention rates reported in earlier studies, it is unclear whether these programs allow visa trainees to circumvent the restrictions (i.e. limited residency seats) that limit the entry of other (non-visa) IMG into the Canadian physician workforce. From a source country perspective, it is unclear whether these programs contribute to ‘brain drain’ (i.e. the loss) of physicians from the source country workforce.

Visa trainees in residency programs represent an under-researched source of physicians contributing to Canada’s overall physician supply. We know little about which physicians are likely to remain in Canada and what factors contribute to their retention. These data are needed to improve the accuracy of physician workforce projections and the transparency of the Canadian PGME system. We hypothesize that visa trainees with Canadian funding sources are more likely to immigrate, given their financial relationship with a Canadian entity.

## Methods

### Aim and study design

Using a cohort design, we followed visa trainees from their exit from PGME through to 2017 using annual files (from 2005 to 2017) of Scott’s Medical Database (SMD). We linked data from the Canadian Post-M.D. Education Registry (CAPER) with SMD. Each faculty of medicine submits data annually on all trainees in PGME programs in Canada to CAPER to create a census of PGME trainees [11]. The submissions, as well as faculty-specific annual census reports, are verified by each faculty of medicine to ensure the data are valid and missing data are minimal. The use of universal administrative data that are reported and verified by all medical schools limits potential sample selection bias. CAPER has created uniform variable definitions to facilitate comparisons across jurisdictions and has linked the records of individual trainees over the course of their PGME training and assigned each trainee a unique identifier. SMD is the most comprehensive data source available to track physician practice locations [12, 13].

Using LinkageWiz v5.5, we used probabilistic matching to link CAPER and SMD data using the following variables: first name, last name, gender, country of medical degree (MD) graduation, university of MD graduation, specialty field, and year of MD graduation. Several combinations of matching variables and weights were tested to identify the best approach (determined by matching scores and visual inspection). Given the variation in text-based fields (e.g. order and spelling of first and last names), we ultimately chose the most readily replicable approach to minimize false positive matches. This approach yields a conservative estimate of trainee retention because the outcome variable (work in Canada) is derived from the match. CAPER census data year covers an academic year and is reported in November of each year [11]. SMD covers each calendar year, but has been reported at different times from year to year. To ensure that trainees did not fall through “data reporting gaps”, we allowed for a minimum of one year overlap to make sure we captured trainees who remained in Canada.

### Setting and participants

Eligible trainees included in the cohort had a “visa trainee” legal status in their first year of training, started their residency program no earlier than 2000, and exited PGME training between 2006 and 2016 (to allow a minimum of one year of follow up). In addition, we only included trainees whose data had a complete record of their source of funding for each year of their post-graduate training.

### Analysis

Using SPSS, after describing the characteristics of the sample, we used Cox regression to compare the retention of visa trainees funded by Canadian, foreign, and mixed sources. The outcome variable (no—never left Canada; yes—left Canada) was based on whether the trainee was continuously present in Canada in each follow-up year after their PGME exit. Trainees who returned to Canada after leaving were coded as having left Canada (that is, we did not look at subsequent entries into Canada). The independent variable, sources of funding over all years of post-graduate medical training (including fellowship, if applicable), was coded as Canadian sources (provincial or federal government, or other public sources; other sources such as charitable organizations, clinical training sites, Canadian business or industries); foreign sources (governments, universities, health care institutions, foundations or industries, and international organizations); or mixed sources (combination of Canadian and foreign) [11]. Covariates included self-reported gender (male/female) at PGME exit, legal status at PGME exit (i.e. now a Canadian citizen/permanent resident), source country (i.e. country of medical school graduation), continuous training (whether there were breaks of more than 1 year), fellowship training (no/yes), program at PGME exit (e.g. family medicine), region of residency training (e.g. Atlantic Canada), year of PGME exit, age at PGME exit, and total years of PGME training in Canada. Variables related to training (e.g. program, region) were based on the final year of training. Medical degree country group was coded as: Canada, “Western” countries (UK, Ireland, Western Europe, US, and Australia); Middle East and North Africa; Other Africa; Asia; Eastern Europe; the Caribbean and South America; and South Africa. These groupings were created, in consultation with knowledge users (e.g. PGME administrators), by considering cultural similarity and group size, and are consistent with definitions used in other studies of IMG in Canada [7], and allow direct comparison across studies.

Potential covariates for the Cox regression were selected on the basis of bivariate analyses (chi-square tests or t-tests). We retained variables in the model if they were significant (*p* < 0.05, based on the Wald test and change in -2 log likelihood value) [14]. Cox regression in SPSS uses the partial likelihood estimation method. In our final regression model, we re-coded the MD source country into three groups (Canada, Western and other) to increase statistical power, since there were no significant difference between trainees from the Middle East and North Africa, Other Africa, Asia, Eastern Europe, the Caribbean and South America, and South Africa. We inspected the survival curves of each significant predictor in the final regression model to verify that they did not overlap and to ensure that the assumption of proportionality of hazards was met [14, 15].

## Results

### Sample characteristics

There were 1916 visa trainees who started a residency program in Canada since 2000 and exited post-graduate training between 2006 and 2016. We excluded three trainees who did not have complete funding data, leaving a study sample of 1913 visa trainees. Of these 1913 visa trainees, 16% remained in Canada during the follow-up period (Table 1). Most trainees were funded by foreign sources (70.7%), graduated from medical school in Middle Eastern and North African countries (70.6%), trained in medical clinical specialties (60.7%), and trained in Quebec or Ontario (70.8%). We found no differences between male and female trainees in terms of their funding source, country of medical school graduation, or any other variables of interest.

**Table 1:** Characteristics of study sample

	Total sample (*n* = 1913) *n* (%)		Total sample (*n* = 1913) *n* (%)
Left Canada after PGME*		Funding—All years	
No—stayed in Canada (never left)	307 (16.0%)	All Years Canadian Sources	431 (22.5%)
Yes—left Canada	1606 (84.0%)	All Years Foreign Sources	1353 (70.7%)
Gender		All Years Mixed	129 (6.7%)
Male	1394 (72.9%)	Did fellowship	
Female	519 (27.1%)	No	968 (50.6%)
Legal status at PGME exit		Yes—after residency	752 (39.3%)
CCPR	282 (14.7%)	Yes—before residency	193 (10.1%)
Visa Trainee	1631 (85.3%)	Region of PGME Exit	
MD Country		BC	137 (7.2%)
Canada	96 (5.0%)	Prairie (AB, SK, MB)	351 (18.3%)
Western	265 (13.9%)	ON	778 (40.7%)
Eastern Europe	22 (1.2%)	QC	575 (30.1%)
South America & Caribbean	56 (2.9%)	Atlantic (NS, NL)	72 (3.8%)
Asia	97(5.1%)	Year PGME Exit	
Africa	20 (1.0%)	2006	102 (5.3%)
Middle East & North Africa	1350 (70.6%)	2007	164 (8.6%)
South Africa	7 (0.4%)	2008	190 (9.9%)
Continuous training		2009	202 (10.6%)
Yes—no absence	1844 (96.4%)	2010	201 (10.5%)
No—1 + year absence	69 (3.6%)	2011	193 (10.1%)
Program at PGME Exit		2012	176 (9.2%)
Family Medicine	67 (3.5%)	2013	170 (8.9%)
Medical Clinical Speciality	1162 (60.7%)	2014	176 (9.2%)
Laboratory Clinical Specialty	92 (4.8%)	2015	165 (8.6%)
Surgical Specialty	592 (30.9%)	2016	174 (9.1%)

	Mean (sd)
Years in Canada after PGME Age at PGME exit Number of years in training	1.41, 2.53 35.31 (4.07) 5.32 (2.14)

*PGME* post-graduate medical education, *CCPR* Canadian citizen/permanent resident, *MD* medical degree, *sd* standard deviation, *BC* British Columbia, *AB* Alberta, *SK* Saskatchewan, *MB* Manitoba, *ON* Ontario, *QC* Québec; *NS* Nova Scotia, *NL* Newfoundland and Labrador

### Bivariate and multivariable analysis for whole sample

At the bivariate level, compared to visa trainees who remained in Canada, a larger proportion of visa trainees who left Canada were funded by foreign sources (76.3 vs. 41.4%), had not changed their legal status (i.e., were still visa trainees; 90.9 vs. 55.7%), had graduated from medical school in Western and Middle East and North African countries (15.0 and 72.2 vs. 7.8 and 61.9%, respectively), had not done any fellowship training (53.2 vs 37.1%), had trained in lab or surgical specialties (4.9 and 32.2% vs. 4.2% and 24.4%, respectively), and trained in BC or Quebec (7.6 and 32.9% vs. 4.9 and 15.3%, respectively) (Table 2).

**Table 2:** Characteristics of visa trainees who did and did not leave Canada following training,

	Full sample (*n* = 1913)		
	Left Canada (*n* = 1606) *n* (%)	Stayed In Canada (*n* = 307) *n* (%)	*P* value
Gender			0.246
Male	1162 (72.4%)	232 (75.6%)	
Female	444 (27.6%)	75 (24.4%)	
Legal status at PGME exit			<0.001
CCPR	146 (9.1%)	136 (44.3%)	
Visa trainee	1460 (90.9%)	171 (55.7%)	
MD Country			<0.001
Canada	67 (4.2%)	29 (9.4%)	
Western	241 (15.0%)	24 (7.8%)	
Eastern Europe	17 (1.1%)	5 (1.6%)	
South America & Caribbean	36 (2.2%)	20 (6.5%)	
Asia	69 (4.3%)	28 (9.1%)	
Africa	13 (0.8%)	7 (2.3%)	
Middle East & North Africa	1160 (72.2%)	190 (61.9%)	
South Africa	3 (0.2%)	4 (1.3%)	
Continuous training			< 0.001
Yes—no absence	1561 (97.2%)	283 (92.2%)	
No—1 + year absence	45 (2.8%)	24 (7.8%)	
Program at PGME Exit			< 0.006
Family Medicine	49 (3.1%)	18 (5.9%)	
Medical Clinical Speciality	961 (59.8%)	201 (65.5%)	
Lab Clinical Specialty	79 (4.9%)	13 (4.2%)	
Surgical Specialty	517 (32.2%)	75 (24.4%)	
Funding—All years			< 0.001
All Years Canadian Sources	304 (18.9%)	127 (41.4%)	
All Years Foreign Sources	1226 (76.3%)	127 (41.4%)	
All Years Mixed	76 (4.7%)	53 (17.3%)	
Did fellowship			< 0.001
No	854 (53.2%)	114 (37.1%)	
Yes—after residency	634 (39.5%)	118 (38.4%)	
Yes—before residency	118 (7.3%)	75 (24.4%)	
Region of PGME Exit			< 0.001
BC	122 (7.6%)	15 (4.9%)	
Prairie (AB, SK, MB)	279 (17.4%)	72 (23.5%)	
ON	617 (38.4%)	161 (52.4%)	
QC	528 (32.9%)	47 (15.3%)	
Atlantic (NS, NL)	60 (3.7%)	12 (3.9%)	
Year PGME Exit			< 0.001
2006	91 (5.7%)	11 (3.6%)	
2007	146 (9.1%)	18 (5.9%)	
2008	177 (11.0%)	13 (4.2%)	
2009	172 (10.7%)	30 (9.8%)	
2010	189 (11.8%)	12 (3.9%)	
2011	171 (10.6%)	22 (7.2%)	
2012	140 (8.7%)	36 (11.7%)	
2013	137 (8.5%)	33 (10.7%)	
2014	139 (8.7%)	37 (12.1%)	
2015	127 (7.9%)	38 (12.4%)	
2016	117 (7.3%)	57 (18.6%)	

	Mean (sd)	Mean (sd)	*P* value
Years in Canada after PGME Age at PGME exit Number of years in training	0.79 (1.86) 34.92 (3.74) 5.17 (2.10)	4.66 (3.02) 37.33 (5.01) 6.15 (2.14)	< 0.001 < 0.001 < 0.001

*PGME* post-graduate medical education, *CCPR* Canadian citizen/permanent resident, *MD* medical degree, *sd* standard deviation, *BC* British Columbia, *AB* Alberta, *SK* Saskatchewan, *MB* Manitoba, *ON* Ontario*QC* – Québec, *NS* Nova Scotia, *NL* Newfoundland and Labrador

**Fig. 2:**
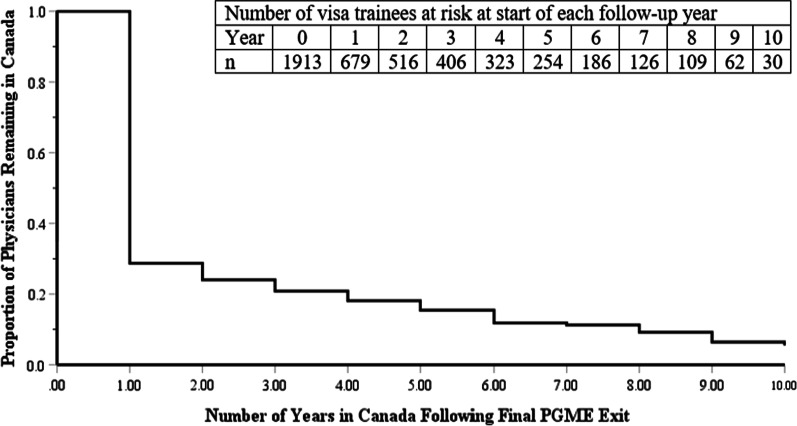
Cox regression survival plot of physicians retention in Canada following their exit from PGME training

**Fig. 3:**
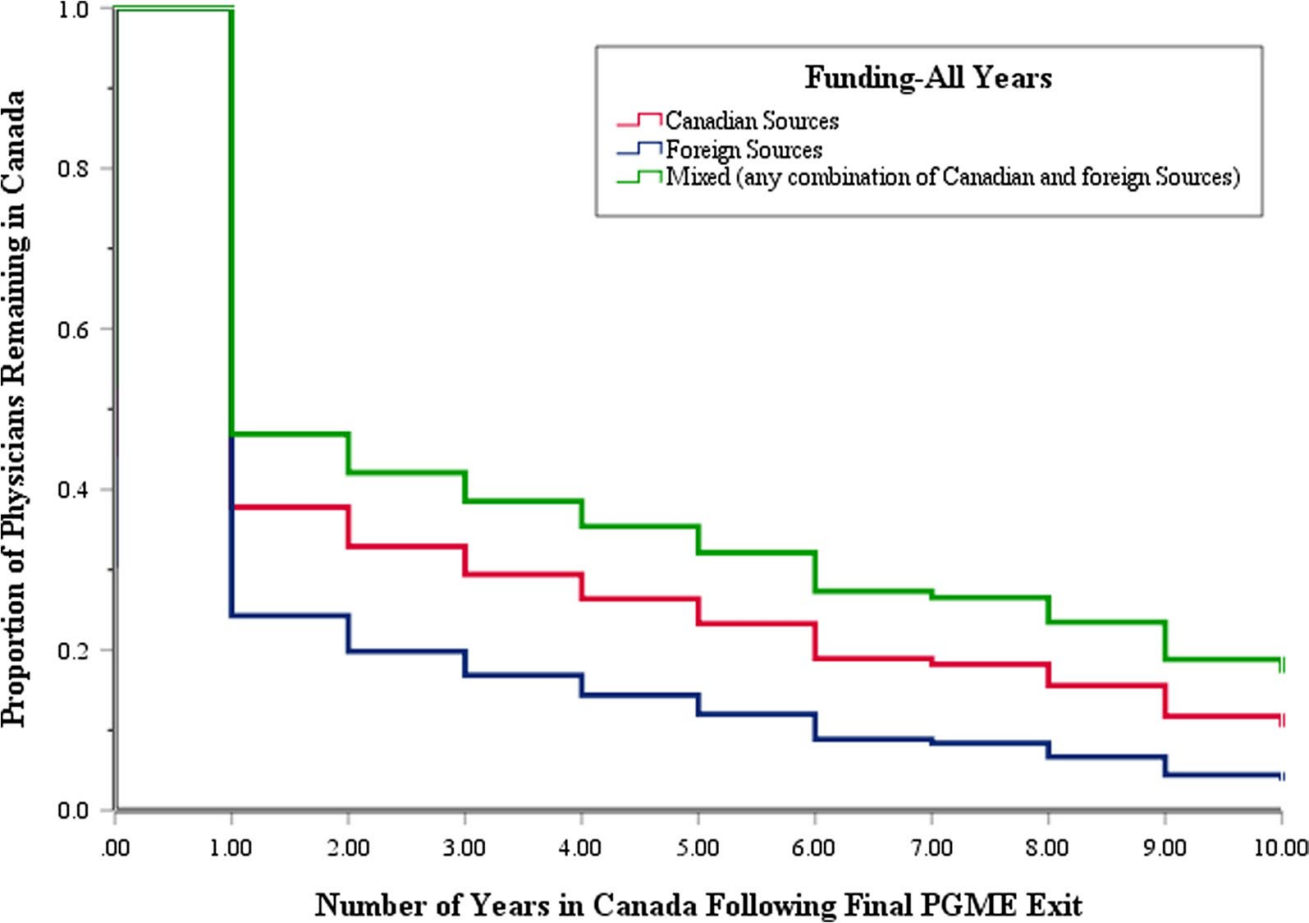
Cox regression survival plot of physician retention in Canada by sources of PGME training funding

Most visa trainees leave Canada following their training. The proportion of trainees remaining in Canada decreases over time, with 35.5% (679/1913); 17.7% (186/1052); 10.8% (11/102) in Canada one, five, and ten years, respectively after their exit from PGME training (Fig. 2). After adjusting for other factors, trainees whose training was funded entirely by foreign sources were more likely to leave than trainees whose training was funded by Canadian sources (Table 3, Fig. 3). Trainees who remained on visas were more likely to leave Canada than those who became a Canadian citizen/permanent resident. Trainees who had graduated from medical schools in Western countries were more likely to leave than visa trainees who graduated from medical school in Canada.

**Table 3:** Cox regression: predictors of visa trainees leaving Canada after PGME training

Predictor	Full sample (*n* = 1913)
	HR (95% CI)
Funding—All years	
All Years Canadian Sources	1.00
All Years Foreign Sources	1.46 (1.25–1.69)
All Years Mixed	0.78 (0.60–1.01)
Legal status at PGME exit	
Canadian/permanent resident	1.00
Visa trainee	1.46 (1.25–1.69)
MD Country Group	
Canada	1.00
Western	1.39 (1.06–1.84)
Other	1.02 (0.78–1.34)

*HR* hazards ratio, *95% CI* 95% confidence interval, *PGME* post-graduate medical education

## Discussion

The proportion of visa trainees in residency programs who remained in Canada after completing their training decreases over time from 35.5% (679/1913) after 1 year to 10.8% (11/102) after ten years of follow-up. The retention rate is lower than previous studies that looked at retention of visa trainees in residency programs using cross-sectional study designs [7, 8], suggesting that trainees may later return to work in Canada after leaving Canada following their PGME training. These figures exclude visa trainees who return to Canada as locums [16], since they are unlikely to be captured in Scott’s Medical Database. Understanding the various pathways, such as visa training programs, through which IMG join the Canadian workforce enhances physician workforce forecasting models and transparency of the Canadian PGME system. It also provides some measure of the impact of these programs on emigration of physicians from source countries.

As hypothesized, residents whose training was funded entirely from foreign sources were more likely to leave than residents who had been funded by Canadian sources or a mix of sources. Over two-thirds of the residents were funded exclusively by foreign sources, highlighting the role of foreign investment in Canada’s PGME system. While some of these funds offset trainee salaries, more research is needed to understand how funds from program fees and tuition support are used in medical schools and academic health centres [17]. Over one-fifth of visa trainees were funded by Canadian sources such as provincial or federal governments, charitable organizations, clinical training sites, and Canadian business or industries. A number of reports have called for greater access to PGME training for the many otherwise qualified Canadian citizen/permanent resident IMG who are unable to secure a residency position [2, 18–20]. These findings identify one potential way of expanding the number of training seats available to Canadian citizen/permanent resident IMG who want to practice medicine in Canada. Rather than recruiting from abroad, these funders and training programs should first consider Canadian citizen/permanent resident IMG, given the numbers who do not match to a residency training program.

Residents who had graduated from medical schools in ‘Western Countries’ were more likely to leave than those who had graduated from medical schools in other countries. Western countries, such Australia, have credential recognition policies that accept Canadian PGME training, and make training in Canada an attractive option for visa trainees from these countries [21]. Canadian students who studied medicine abroad are not included in this group, since they are Canadian citizens and permanent residents and can work and study in Canada without visas.

Female trainees comprised just over one-quarter of the study sample. Women comprise 53% of Canadian and permanent resident PGME trainees (both CMG and IMG) [4]. The smaller numbers of women in our sample is consistent with previous studies of visa trainees [7] and reflects the concentration of visa trainees in specialist programs (which traditionally attract more male trainees).

For Canadian policy makers, the study results suggest that the visa trainee residency program is addressing its original goals of meeting Canadian training program needs, providing services, and fulfilling Canada’s obligation to support medical training in less developed countries [6, 7, 10]. For policy makers outside Canada, these results show visa trainee residency programs in Canada does not lead to a “brain drain” of source country physicians, in the short-term. Further research, from the perspectives of trainees and source countries, is needed to understand the factors that contribute to the return and retention of visa trainees to the source country to ensure that these training investments ultimately improve the source country physician workforce in the long term.

### Limitations

We purposefully used the most readily replicable linking strategy that produces a conservative estimate of retention; true retention rates are likely higher. Moreover, our matching approach relies on naming approaches (e.g., order of first and last names, last name equals family name, etc.) that may be better understood Western Countries, and translation of names into English or French (i.e., Canada’s official languages). Hence, we may disproportionately underestimate retention rates of trainees from non-Western, non-English speaking countries, who may be less likely to match to the SMD, despite remaining in Canada. While CAPER data capture Canadian legal status (i.e. Canadian citizen or permanent resident), it does not describe citizenship for visa trainees. Citizens from certain countries (e.g. Saudi Arabia) may graduate from medical schools from those countries. Moreover, while CAPER data capture change in legal status during PGME training (i.e. allowing us to identify those who became Canadian citizens or permanent residents during training), we are unable to identify changes in legal status during the follow-up period. Some visa trainees may return to Canada after initially leaving Canada, however, since these individuals are no longer in PGME, their legal status is not captured in the available data (i.e. SMD or CAPER). We are unable to identify what programs (e.g. immigration, temporary foreign worker) were used to facilitate visa trainees’ subsequent entry into Canada, or long-term stay in Canada.

## Conclusions

The proportion of visa trainees remaining in Canada decreased over time, from 35.5% (679/1913) to 17.7% (186/1052) to 10.8% (11/102) one, five, and ten years, respectively, after their exit from PGME training. Visa trainees with more Canadian connections (such as funding from Canadian sources or changes in legal status to permanent resident) are more likely to remain in Canada. Almost two thirds of visa trainees in residency programs are funded by foreign sources, illustrating the role of foreign investment in Canada’s medical education system.

## Data Availability

The data that support the findings of this study are available from CAPER but restrictions apply to the availability of these data, which were used under license for the current study, and so are not publicly available. Data are however available from the authors upon reasonable request and with permission of CAPER.

## Abbreviations

PGME: Post-graduate medical education
IMG: International medical graduate
CMG: Canadian medical graduate
SMD: Scott’s Medical Database
CAPER: Canadian Post-MD Education Registry
MD: Medical degree
